# Dignitary Medicine: A Novel Area of Medical Training

**DOI:** 10.7759/cureus.5962

**Published:** 2019-10-22

**Authors:** Mobarak A Al Mulhim, Robert G Darling, Hetaf Kamal, Amalia Voskanyan, Gregory Ciottone

**Affiliations:** 1 Emergency Medicine, Beth Israel Deaconess Medical Center, Boston , USA; 2 Emergency Medicine, Uniformed Services University of the Health Sciences, Bethesda, USA; 3 Emergency Medicine, Beth Israel Deaconess Medical Center, Boston, USA

**Keywords:** dignitary medicine, presidential health, curriculum development research, concierge medicine, vip, protective medicine, emergency medicine, curriculum, curriculum development

## Abstract

Dignitary medicine (DM) involves the provision of healthcare to government leaders and other high-profile individuals collectively referred to as “dignitaries.” Due to the unique circumstances around their lifestyle, dignitaries often receive suboptimal healthcare. We define the requisite skills needed to practice DM based on the available literature and provide a framework for training providers in these skills.

A review of the English language medical literature focussing on adult subjects was performed, searching for terms such as “dignitary medicine,” “VIP medicine,” and “protective medicine.” Literature was gathered from CINAHL, Google Scholar, PubMed, EBSCOHost, and San Bernardino County Library databases and then analyzed by experienced DM providers.

A total of 23 relevant articles were eligible for review. No meta-analyses on the subject exist. We found that existing studies highlight skills in wellness, executive health, and protective medicine, which form the backbone of DM.

The burgeoning field of DM encompasses several disciplines and skills. We strongly recommend a structured curriculum for the field of DM, focused on dignitary wellness, executive health, and protective medicine.

## Introduction and background

Dignitaries are a select group of high-profile politicians, royal family members, and businessmen. While similar to celebrities, athletes, and musicians, dignitaries are not only famous, but their health can impact the political and economic health of a city, nation, or even the world. As such, dignitaries have a unique set of healthcare needs, which the general public does not have [1]: they require aggressive preventive and protective medical care, 24/7 high-quality personalized healthcare, and an even greater degree of privacy than ordinary citizens. International travel, especially as part of humanitarian or political missions, can create novel exposures to infections or illnesses. Medical and transportation infrastructure may be primitive on many trips, creating logistical challenges the medical team must plan for and overcome [1]. Threats of assassination add another layer of concern for dignitaries. The average person traveling on a business trip will be well-suited to receive emergency care at a local ED; however, if the president of a nation merely arrives in an emergency department (ED) and is not properly cared for, an international calamity may ensue. Communication with the press, media, and public about the condition of a dignitary takes on an added dimension, which average patients just do not require. Furthermore, privacy is of even greater concern for dignitaries than the average patient.

Dignitaries often have a close circle of advisors and confidants that limit or hinder the doctor-patient relationship [2]. The power, prestige, and station that a dignitary has may result in some providers being awestruck and not providing the same advice or treatment suggestions as to a non-dignitary patient [3]. A dignitary may be a critical member of an international delegation and illness may threaten the purpose of an international business or political trip. On the flip side, dignitaries often receive worse care than the average patient because doctors and care teams may disrupt the normal and smooth flow of clinical operations to handle such patients [2]. Furthermore, the "Chairperson Syndrome" [2] may emerge, where the most senior members of a department may insert themselves into the care team while other members of the team may be better suited and skilled to manage the clinical issues at hand. The complex schedules and demands on dignitaries also make providing care a challenge.

To combat the deficiencies in the care provided to dignitaries, we propose a novel field of medical training, “Dignitary Medicine” (DM), and offer suggestions on the core competencies of DM trainees. We analyzed the existing literature to inform the needed training and competencies and report the results of our investigation herein.

## Review

A unstructured review of the English language medical literature focused on adult subjects was performed. The source of information involved collecting data from EMBASE, CINAHL Plus (Cumulative Index of Nursing and Allied Health Literature Plus), Cochrane Library, PubMed, Google Scholar, Global Health Library, SBC Library (San Bernardino County Library), and EBSCO Host (Elton B Stephens Company Host) databases. The databases were searched for the terms “dignitary medicine,” “executive health,” “concierge medicine,” “protective medicine,” “royal health,” “presidential health,” “royal physicians,” “presidential physician,” “the WHMU” and “the royal clinics.” Furthermore, two books “When Illness Strikes the Leader" written by Post & Robins in 1995 and "The White House Physician: A History from Washington to George W. Bush," written by McFarland Deppisch in 2007 were evaluated. Institutional review board (IRB) approval was not needed, as human subjects were not involved in this project.

The inclusion criteria included articles reviewed in English and published in peer-reviewed journals. The articles included dignitaries as a patient population, their respective physicians, or practice related to those populations. Articles addressing politics, news reports, advertisements, and health care professionals other than a physician were excluded.

Two team members reviewed each of the abstracts and the related titles. Studies that were marked for potential inclusion by both reviewers underwent a full-text review by two separate team members. If we agreed that a given study did not merit inclusion, the study was excluded. In instances where the reviewers did not agree, the disagreement was resolved through consensus and discussion with additional team members.

We extracted the following information from the potential articles: publication details (authors, publication type, title, journal, volume, issue, page numbers, publication location, and digital object identifier (DOI)); study details (timeframe, location of study, and study design); participant information (source population, definition of the target, target population size, and source of population size); and other salient data (interventions, the core skills of DM, and best principles).

**Figure 1:**
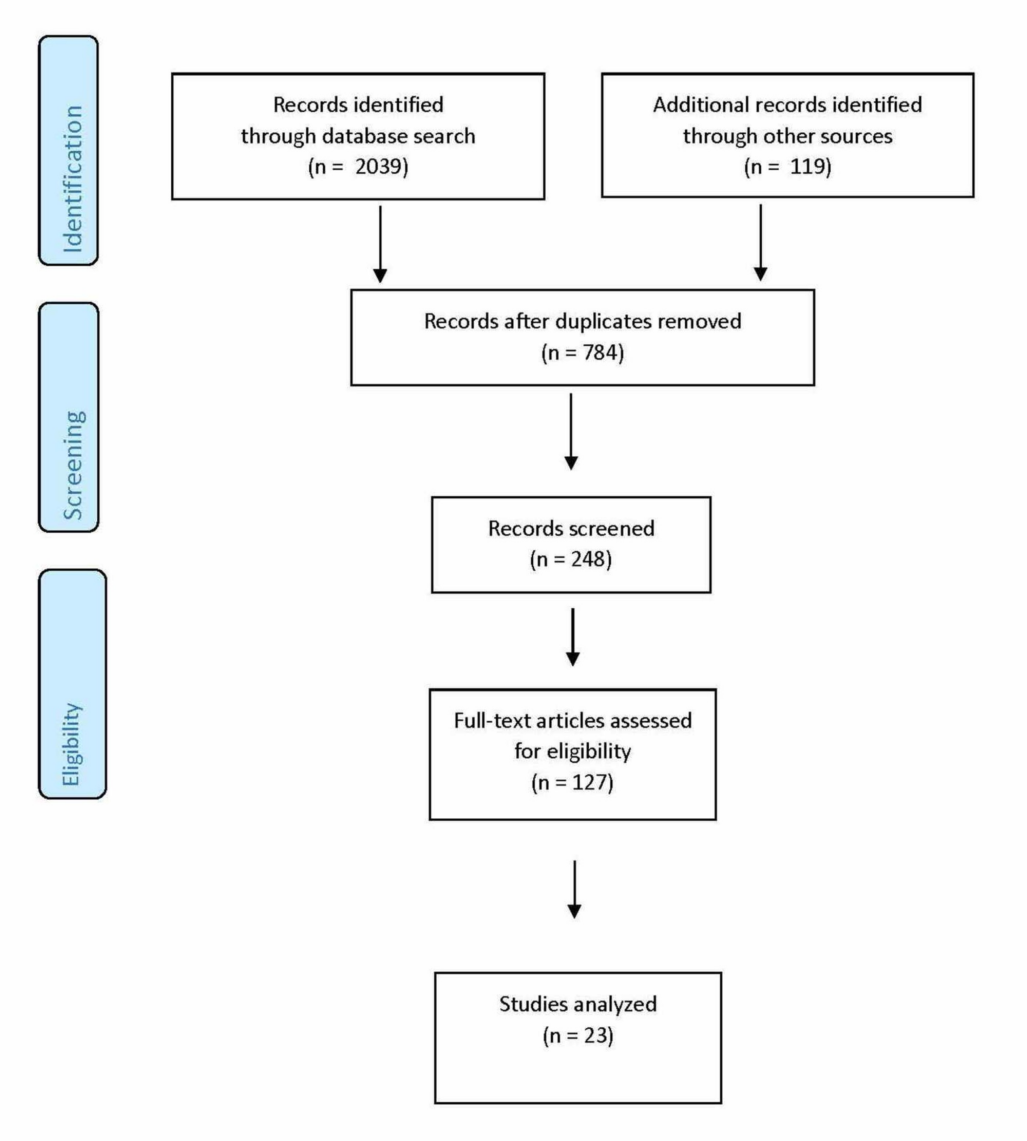
Search strategy and flowchart

We found 2039 articles in our initial search but most were of poor quality and did not meet inclusion criteria (Figure 1). We only found 23 articles at the end of our search string that met inclusion criteria. Based upon our review of the included studies, we identified four recurring themes in the literature: executive health, protective medicine, overall health/wellness, and leadership/professionalism/ communication (Table 1). The existing literature covers these skills, with an emphasis on executive health and protective medicine, with several studies focusing on specific events such as the attempted assassination of President Reagan.

**Table 1:** Articles grouped by relevant DM skill grouping EH = Executive Health PH = Protective Health L = Leadership W = Wellness P = Professionalism

Author	Year	Study Population	Key Finding	Skill Areas
Aaron et al [4]	1994	US President	Emphasis on ready access to specialty care	EH, PH, L
Ahmed et al [5]	2018	US President	Treating the VIP the same as other patients	EH, L
Alfandre et al [6]	2016	VIP’s	Treating the VIP the same as other patients	EH, L
Band et al [1]	2012	VIP’s	Emphasis on pre-hospital and tactical skills	PH
Block at al [7]	1993	VIP’s	Awareness of treating VIP’s differently than other patients	EH, L
Dalen at al [8]	2017	Concierge Practice	Basics of concierge medicine	EH
Diekma [9]	2000	VIP’s	Approach to VIP patients in the ED	EH
Diekma [10]	1996	VIP’s	Approach to VIP patients in the ED	EH
Eddy [11]	2016	Healthcare teams	Best practices for healthcare teams	L
Edlich et al [12]	2004	US President	Emergency medical systems	PH
Feuer et al [3]	1978	VIP’s	Impact of VIP’s on a medical team	EH, L
Friedman [13]	1995	US President	Disability and the US president	W
Groves et al [14]	2002	VIP’s	Psychiatric care of VIP’s	EH, W
Guzman et al [2]	2011	VIP’s	Nine key principles to managing VIP patients	EH, L
Lerner [15]	2001	First Lady	Eleanor Roosevelt and a possible missed diagnosis	EH, W
Mahmood et al [16]	2014	Physician patients	Framingham Heart Study	W
Martin et al [17]	2004	VIP’s	VIP psychiatric health	EH, W
Schenkenberg et al [18]	2007	VIP’s	Ethics and VIP health	EH, L
Silverman et al [19]	2012	VIP’s	VIP’s and psychiatric care	EH, L
Smith and Shesser [20]	1988	VIP’s	Emergency care of VIP’s	EH, P
Strange [21]	1980	VIP’s	VIP’s with illness	EH, P
Weintraub [22]	2012	VIP’s	VIP’s and mental health	EH

**Figure 2:**
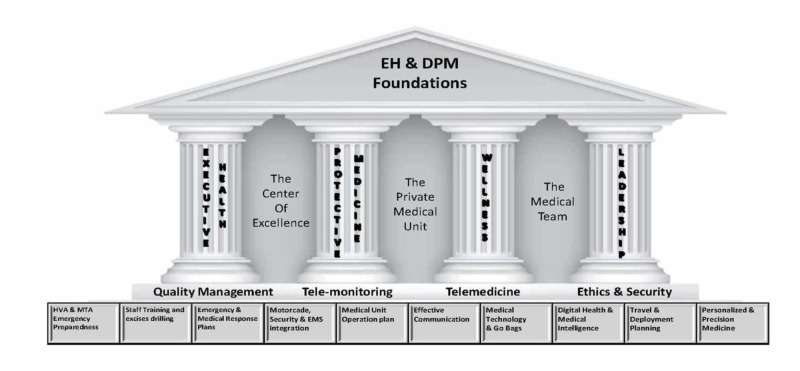
The foundations of dignitary medicine

To offer a broad and full-service array of medical services for dignitaries, as opposed to a fragmented approach delivered by a slew of specialists, we believe DM physicians need mastery of executive health, protective medicine, overall wellness, and, finally, professionalism, leadership, and communication (Figure 2). These four thematic areas emerged in the scant literature available for review on this topic and form a starting point for further curricular development and, potentially, training in this field. We further define these unique skills below.

Executive health refers to the general health maintenance of the dignitary, something akin to concierge medicine [23]. DM physicians provide some primary care but may also refer specific health matters to specialists in predesignated medical centers of excellence. The DM physician commonly establishes a “hotline” system by arranging immediate access to the best available specialty services and consultants around the world, with particular attention to medical record security and concierge access.

Protective medicine involves all protective, tactical, and emergency medical care for the dignitary, his or her immediate family, and entourage, with a strong focus on preventing injury, illness, and even assassination. Procedures related to delivering protective care during travel and alongside motorcade activity are essential aspects of protective medicine. To be the most effective, protective medical care requires high levels of risk assessment and mitigation procedures, utilizing the best security and intelligence resources in collaboration with the dignitary’s other support services. As such, non-medical personnel, such as security forces and the administrative staff of the dignitary, are critical partners for successful protective medical care.

Wellness involves the overall health and wellbeing of the dignitary. The DM physician designs a personalized health care plan that promotes primary prevention, with the goal of increasing longevity and promoting an illness-free life. This requires utilizing the best available evidence-based practices, screenings, and lifestyle interventions.

The leadership of a complex, multi-disciplinary team is requisite in this role. The DM physician is expected to oversee a complex medical team, uphold the highest level of quality, and be abreast of and implement advances in the care of the dignitary. Further, communication with the media, other providers, politicians, and security staff is a critical aspect of the DM physician’s role. Finally, a good DM physician leader exercises the utmost in professionalism in safeguarding the privacy of the medical affairs of the dignitary.

Throughout history, dignitaries have found it challenging to receive high-quality healthcare while, at the same time, maintaining their unique requirements for security and privacy [24-25]. Both assassination and the natural death of a governmental leader can threaten stability within the countries they serve [5]. The societal disruptions caused by the deaths of President Harrison due to pneumonia, President Harding from myocardial infarction, and President Tayler from gastroenteritis, as well as the assassinations of Presidents Garfield, Kennedy, Lincoln, and McKinley, have led to the understanding that the President's health and protection has become a fundamental national security issue [26]. As such, the DM physician’s role goes beyond that of a doctor and patient and is a factor in national and geo-political stability.

Additionally, with such a diverse array of issues that require expert medical care, it is clear the DM providers need specialized training and skills in order to best care for dignitaries. In some cases, victims of assassination may benefit from a DM physician skilled in protective medicine and trauma care. In the event of illness, secrecy around a dignitary’s condition may have beneficial effects for the patient and society at large. These patients demand high-level security and confidentiality when being treated at a hospital and such skills will need to be taught to DM providers [27].

In addition, there are a number of key clinical principles that allow the optimal care of the dignitary while not becoming awestruck and maintaining professionalism [15]. Silverman and his colleagues present literature demonstrating that physicians assigned to dignitaries may fail to provide the desired healthcare services, owing to the pressure associated with the status of dignitaries [28]. He goes on to offer possible solutions, essentially suggesting the treatment team includes DM physicians [28].

It is essential that physicians and medical personnel tasked with the provision of healthcare to dignitaries are sufficiently trained. Such training aims to ensure that the physicians within this area of medicine are equipped with the necessary skills to provide for the wellbeing and protection of dignitaries from illness and other health threats. To accomplish this, several options exist, including post-residency specialized fellowship training. Training may also be achieved through practice standardization and development of a formal curriculum and guidelines for DM. This professionalization, as well as standardization of such healthcare models, will help in the provision of optimized, high-quality care to dignitaries and their family members.

## Conclusions

This review shows that the existing literature on DM is fragmented and no single article synthesizes the required skills into one review. We believe our paper can serve as an excellent resource for providers in this field. Given the combination of skills needed to practice DM - executive health, protective medicine, wellness, and leadership - we believe this is a unique area of medical training and that this review can serve as the basis of future training.
